# Orthostatic intolerance with small heart and/or disequilibrium in patients with myalgic encephalomyelitis/chronic fatigue syndrome: clinical update and paradigm shift

**DOI:** 10.3389/fmed.2026.1744154

**Published:** 2026-07-29

**Authors:** Kunihisa Miwa

**Affiliations:** Miwa Naika Clinic, Toyama, Japan

**Keywords:** chronic fatigue syndrome, disequilibrium, myalgic encephalomyelitis, orthostatic intolerance, postural orthostatic tachycardia syndrome, small heart

## Abstract

Orthostatic intolerance (OI) is characterized by the inability to maintain an upright posture without experiencing severe signs and symptoms, including hypotension, palpitations, light-headedness, pallor, fatigue, weakness, dizziness, impaired concentration, tremulousness, and nausea. The majority of patients with myalgic encephalomyelitis (ME) or chronic fatigue syndrome (CFS) exhibit OI, which is the primary factor restricting daily functional capacity. During an upright posture, approximately 800 mL of blood is translocated from the intrathoracic venous compartment to the veins of the buttocks, pelvis, and legs. Under normal conditions, compensatory cardiovascular responses to this orthostatic stress include activation of the muscle pump through calf muscle contraction and a neurogenically mediated increase in heart rate and in systemic vascular resistance. The majority of the symptoms of OI are believed to be cardiovascular and related to reduced cerebral blood flow and excessive activation of the sympathetic nervous system. However, compensatory sympathetic activation is essential for maintaining orthostasis. Many patients have a small left ventricle and associated low cardiac output. In addition, both the renin–aldosterone and antidiuretic hormone systems that regulate circulatory blood volume were downregulated. Postural stability is necessary for maintaining the static balance critical for performing many daily activities. Recently studies have reported that postural instability or disequilibrium, potentially associated with central vestibular dysfunction, should be considered to be involved in the pathogenesis of OI among patients with ME/CFS. Disequilibrium should be recognized as an important cause of OI in those patients.

## Prologue

### Former chronic fatigue syndrome, now myalgic encephalomyelitis

Chronic fatigue syndrome (CFS), which affects many young people in modern, stressful society, is an important health problem, characterized by persistent, relapsing, and severely disabling fatigue that is not resolved by rest, causing a marked reduction in working activity (1–3). Despite the serious public health burden imposed by CFS, effective diagnostic, treatment, and prevention strategies are still unavailable because the etiology, risk factors, and pathophysiology remain unclear. In 2011, myalgic encephalomyelitis (ME)-associated central nervous system dysfunction was postulated as the primary cause of CFS (4). Several pathobiological mechanisms have been proposed for ME, including redox imbalance or exaggerated oxidative stress, immune abnormalities, central nervous system inflammation or neuroinflammation, energy production/transport impairments, mitochondrial alterations, and endothelial and vascular disturbances (4–11). Recently, these mechanisms have been unified into a common pathophysiological hypothesis of central nervous dysfunction centered on the pathologically reactive neuroglia, primarily microglia and astrocytes, in stress-responsive brain regions, such as the hypothalamic nuclei and other parts of the limbic system. This hypothesis can efficiently explain not only the onset of ME symptoms but also the hallmark features of the disorder, such as post-exertional malaise, as well as its progression over time (12). A wide range of infectious agents, as well as a lot of non-infectious noxious stressors, including surgical or accidental injury and serious psychological damage, such as the experience of a major earthquake, have been suggested to trigger the development of ME, and one such pathogen may be the coronavirus disease 2019 (COVID-19) virus (4, 13–17). To date, no specific biochemical or physiological marker has been established for the diagnosis of ME. Diagnosis of ME can be made only after alternative known medical and psychiatric causes of chronic fatiguing illness have been excluded (4, 18).

## ME/CFS as a low output syndrome due to small heart

### Small heart as a constitutive factor predisposing to myalgic encephalomyelitis

The concept that a heart that is small in relation to the body is inadequate for work was first stated by Laennec (19) in 1826. Later, Master (20) reported several so-called “neurocirculatory asthenia” cases characterized by weakness or fatigue even after ordinary exertion, tachycardia, palpitation, and dyspnea, in patients who had a small heart shadow on chest radiography. The most frequent complaints are fatigue or weakness, rapid heart rate, precordial or chest pain, shortness of breath, nervousness, trembling, sweating, and fainting, many of which resemble those in ME patients. Master hypothesized that these symptoms were caused by diminished venous return, diminished cardiac output, anoxemic heart muscle, and decreased oxygen saturation of the blood due to a congenitally or constitutionally small heart. Similarly, DaCosta (21) described an “irritable heart” in 1871, a peculiar functional disorder of the heart observed in the military population during the American Civil War. The disorder frequently presented either after an episode of diarrhea and persisted even after the digestive disturbances had diminished, or it originated suddenly without previous digestive disorder. Fatigue was an almost universal complaint in DaCosta’s syndrome, although other symptoms included palpitations, cardiac pain, headache, dimness of vision, and giddiness (21, 22). Because diarrhea may lead to dehydration and reduce venous return or preload, resulting in further decreases in stroke volume and cardiac output in subjects with a small heart.

The apparent heart size or cardiothoracic ratio (CTR) is influenced by the position of the heart in the thoracic cage. A small heart may be caused by a standing or dropped heart associated with a low diaphragm and a narrow chest in individuals with a thin physique and reduced fat content in epicardial and pericardial spaces. Consequently, the pathognomonic significance of a small heart has not been established and is often overlooked or even ignored (23).

Patients with “small heart syndrome” (24) are known to have slender structures, with low body mass indices, and frequently exhibit visceral ptosis, including wandering kidney, asthenia, nervousness, and cold feet, suggesting physical, autonomic nervous, and psychological irritability or a lack of relaxation (20, 24–26). Thoracic skeletal abnormalities, such as a shallow chest, a straight thoracic spine, loss of physiologic thoracic kyphosis, and scoliosis, are frequently noted in patients with small heart syndrome (27–29). Mitral valve prolapse (27–30) associated with symptoms such as chest pain, palpitation, and dyspnea is a frequent complication of patients with small heart syndrome (20, 22, 27–29). Many individuals with small heart syndrome appeared to be emotionally sensitive, often delicate and nervous. These conditions may be genetically determined, although several other factors may contribute to the constitution of these patients (20).

To work and perform other duties without excessive exhaustion, patients with small heart syndrome need adequate rest and both physical and emotional relaxation (25, 31). Various triggers, including loss of appetite, diarrhea, and summer sweating, can cause dehydration, resulting in preload reduction. Further reductions in cardiac performance caused by preload reduction may play an important role in predisposing subjects with small heart to symptoms, including general malaise, fatigue, dizziness, orthostatic dysregulation, dyspnea on effort, and palpitations. Autonomic nervous dysfunction, with possible accentuated basal parasympathetic tone, may be associated with these symptoms via inhibition of sympathetic activation, which is required to maintain proper cardiac function (32). Habitual exercise, which can facilitate autonomic nervous system adaptation and induce pulmonary and cardiovascular conditioning, may improve functional work capacity and fatigue by increasing cardiac output. Diarrhea, sweating, and loss of appetite as triggering factors for exacerbation should be avoided or treated properly. Constitutional changes or conversion are not easy to achieve, as patients may need to take holidays occasionally. People in their workplace and society should understand and accommodate their specific needs.

### ME and cardiac dysfunction or low cardiac output

Cardiovascular dysfunction, such as chronic heart failure, can be a major cause of disabling chronic fatigue, and many symptoms observed among ME patients are common in patients with low cardiac output syndrome. Indeed, accumulating evidence points to a possible circulation problem in ME (25, 31–34). The reported findings included autonomic dysfunction (35, 36), lower plasma volume and/or red cell mass (37, 38), and abnormalities in neurohumoral systems of circulatory control (39–41). In 2003, Peckerman et al. (34) provided a preliminary indication of reduced cardiac output in patients with severe CFS.

### Small heart syndrome as an unrecognized cause of ME

Recently, it has been reported that chest roentgenographic, electrocardiographic, and echocardiographic examinations revealed several distinct findings in ME/CFS patients (25, 26, 31). Specifically, a small heart shadow was often observed on the chest roentgenogram in these patients. In 2008, we first reported that “small heart” with low cardiac output demonstrated by both roentgenography and echocardiography is prevalent in CFS patients (25). In this report, small heart syndrome (CTR ≤42%) was significantly more prevalent in the CFS group than in the control group. Narrow chest, orthostatic dizziness, cold feet, right kidney palpability, and mitral valve prolapse were significantly more prevalent in CFS patients with a small heart than in the control group, as well as in CFS patients without a small heart. Echocardiographic evaluation demonstrated impaired cardiac function with low cardiac output due to a small heart in many of the patients with chronic fatigue syndrome. Significantly smaller LV end-diastolic and end-systolic dimensions, stroke volume, and cardiac indexes were observed in CFS with a small heart compared with control subjects with a normal heart size (42% < CTR < 50%). Thus, a considerable proportion of ME patients have a small heart, and cardiac performance may be impaired with low cardiac output due to a small LV chamber size and reduced cardiac function, resulting in low stroke volume and cardiac indexes in many patients (32). In addition, both cardiac size and performance were reduced during the exacerbation phase and improved during the remission phase in CFS patients with “small heart,” suggesting that small heart syndrome with impaired cardiac function may play an important role in the genesis of ME/CFS (31). In addition, CTR increased significantly during the remission phase compared with the exacerbation phase (31). Reduced LV ejection fraction was not observed in any patients, suggesting the absence of myocardial systolic dysfunction. Many ME patients have low cardiac output, and the resulting low-flow circulatory state may make it difficult for patients to meet the demands of everyday activity, and it may also lead to fatigue and other conditions.

Indeed, ME patients had a variety of possible cardiovascular complaints, including chest pain, palpitations, dyspnea or shortness of breath, coldness of feet, dizziness, and fainting, although these symptoms are all not necessarily attributable to cardiovascular dysfunction (32). Frequently noted physical examination findings such as epigastric splash sound, right kidney palpability, cold feet, and pretibial pitting edema may be related to visceral ptosis with slender build and peripheral circulatory impairment (32). Weakness, rapid heartbeat, and orthostatic dizziness may be related to hypotension and orthostatic dysregulation. Auscultatory findings, including a late systolic murmur and a mid-systolic click, suggested typical mitral valve prolapse in some patients. In addition, electrocardiograms showed severe sinus arrhythmia and vertical or right axis deviation in a considerable number of the patients, suggesting parasympathetic predominance and vertical heart position (32). Following our reports, Hurwitz et al. (38) reported that severe CFS patients had lower cardiac output, as indicated in their echocardiographic results, as well as lower total blood volume, plasma volume, and red blood cell volume, as indicated by dual-tag blood volume assessments, compared with patients in the control group, suggesting a comorbid hypovolemic condition. The pathognomonic significance of a small heart should be recognized as a constitutional factor predisposing individuals to low output syndrome (25, 42).

### Orthostatic intolerance (OI) (classical mechanism)

**Figure 1:**
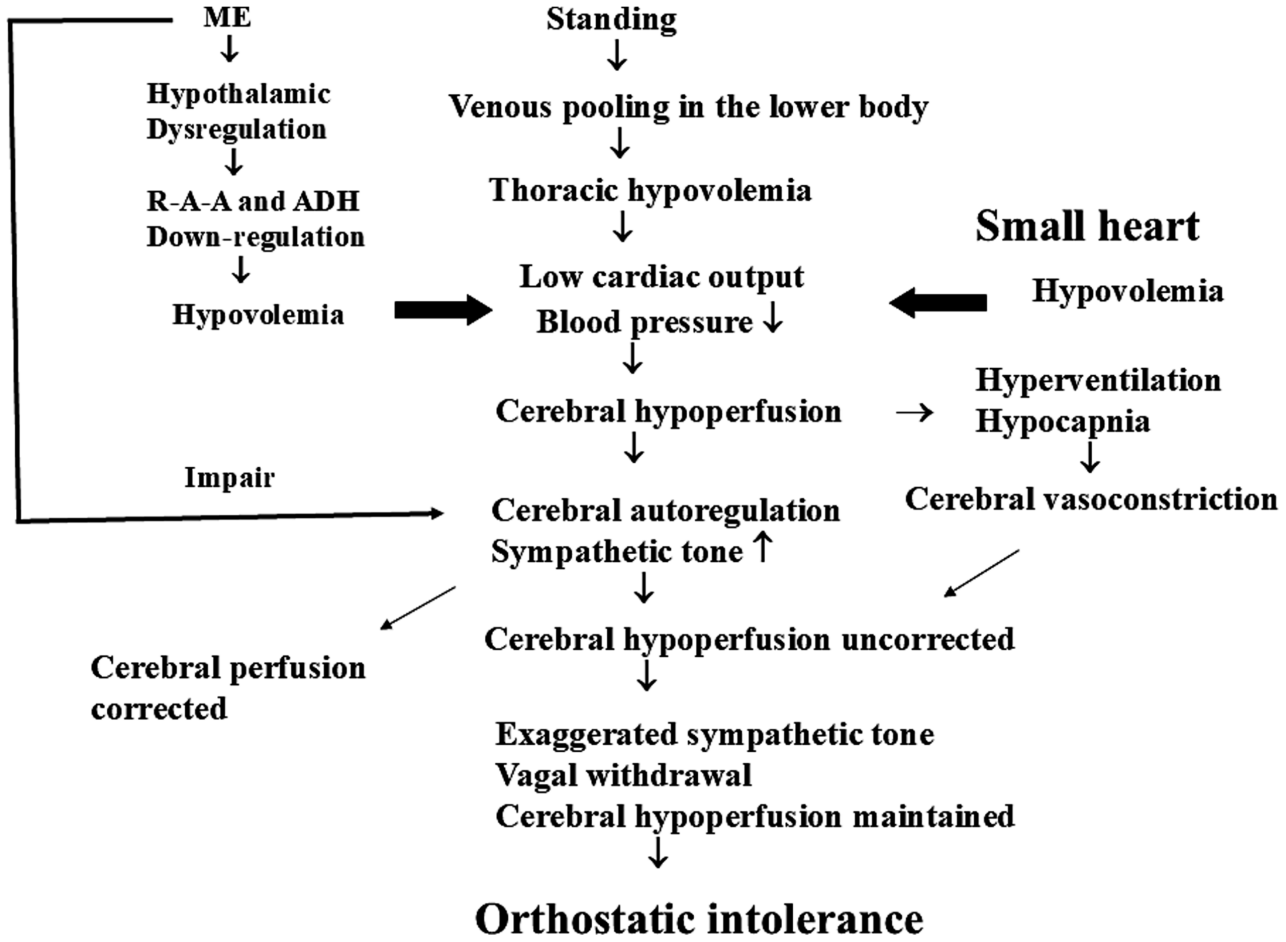
Circulatory mechanism for OI. ME: myalgic encephalomyelitis.

Patients with OI have been clinically recognized (43–45). These patients predictably develop symptoms of disabling fatigue, dizziness, diminished concentration, tremulousness, and nausea while standing. Simple activities such as eating, showering, or low-intensity exercise may profoundly exacerbate these symptoms. Reduced cerebral blood flow with impaired cerebral oxygenation during an upright posture is considered a major mechanism for OI (37, 46). However, compensatory sympathetic activation also appears to play an important role in the development of various symptoms when exaggerated (Figure 1) (44–49).

Assuming an upright posture causes the translocation of approximately 800 mL of blood from the intrathoracic venous compartment to the veins of the buttocks, pelvis, and legs (50). The normal compensatory cardiovascular response to this orthostatic stress includes a couple of major mechanisms. One is muscle pump utilization associated with calf muscle contraction, which starts simultaneously with standing and leads to accentuated venous return from the legs. The other important mechanism is a neurogenically mediated increase in heart rate and in systemic vascular resistance (50). Not all vascular beds contribute equally to the reflex increase in vascular resistance (51). Splanchnic vasoconstriction accounts for one-third, whereas skin and muscle vasoconstriction account for approximately 40% of the increased vascular resistance during normal levels of orthostatic stress (50). Symptoms of orthostatic intolerance develop as these reflexes approach the limit of compensation.

In 1982, Rosen and Cryer were the first to describe a woman with a 7-year history of disabling postural tachycardia and palpitations associated with an idiopathic reduction in plasma volume (43). In 1993, Schondorf and Low (44) reviewed the patients who exhibited exaggerated tachycardia at rest or during head-up tilt and coined the term “idiopathic postural orthostatic tachycardia syndrome (POTS),” which may be a manifestation of a mild form of acute autonomic neuropathy. Studies suggest that postural orthostatic tachycardia syndrome (POTS) is accompanied by a range of autonomic nervous system abnormalities, including vagal withdrawal and enhanced sympathetic modulation, associated with findings consistent with pooling in the lower limbs (44–49, 51). Studies in adolescents also suggest that POTS physiology underlies OI in the majority of CFS patients (37, 48–50). POTS is a frequent finding in patients with CFS (37, 48–50). Clinical evaluation of CFS patients should include response to standing (52).

It has been reported that patients with postural orthostatic tachycardia, which is often observed in patients with chronic OI and/or ME, have a smaller heart coupled with reduced blood volume compared with healthy controls (53, 54). Using cardiac magnetic resonance imaging, Fu et al. (53) precisely assessed heart size, mass, and blood volume in POTS patients and found that all of these parameters were significantly lower compared with healthy sedentary controls. The marked orthostatic tachycardia in these patients appeared to represent a physiologic compensatory response to a smaller stroke volume, and exercise training improved this syndrome in majority of the patients (53, 54). Fu et al. (53) proposed a new term for POTS based on its underlying pathophysiology—the “Grinch syndrome,” because, in Dr. Seuss’s famous children’s book, the main character had a heart that was “two sizes too small.” In their assessment of both sympathetic baroreflex sensitivity and cardiovagal baroreflex sensitivity, the function of the autonomic nervous system was intact in the patients (53). However, other researchers have postulated autonomic nervous system dysfunction, with exaggerated sympathetic nervous activation beyond compensatory levels during standing, as a major mechanism underlying symptoms (43, 44, 48, 49, 51).

### Pathophysiology of OI in ME (classical)

Potential pathophysiological mechanisms in chronic orthostatic intolerance include β-adrenergic hypersensitivity (55), decreased plasma volume (56), inappropriate venous pooling (37), and possible dysautonomia (44–51). Several disorders, including delayed orthostatic hypotension (37), neurally mediated hypotension (57), and postural orthostatic tachycardia syndrome (43–46, 58), may underlie or promote orthostatic intolerance. Delayed orthostatic hypotension can be caused by excessive gravitational venous pooling (37, 59). Impaired vasoconstrictor function with relative bradycardia is often observed in neurally mediated hypotension (57). In 1995, Rowe et al. (57) described cases with demonstrating an overlap between the symptoms of CFS and neurally mediated hypotension, suggesting that neurally mediated hypotension should be considered a treatable cause of CFS. Exaggerated tachycardia and vasoconstriction without hypotension of POTS during standing can cause OI (43–46), although the pathophysiology of POTS remains unclear.

van Campen et al. used extracranial Doppler imaging of the carotid and vertebral arteries, accounting for both flow velocity and vessel diameters, to assess whether orthostatic symptoms in ME/CFS are associated with reduced cerebral blood flow (60–62). These studies indicate that orthostatic intolerance symptoms are related to cerebral blood flow reduction and that the majority of ME/CFS patients show an abnormal cerebral flow reduction during orthostatic stress testing. These findings may have implications for the diagnosis and treatment of ME patients. In ME/CFS patients with orthostatic intolerance symptoms, cardiac output and cerebral blood flow are significantly reduced during a tilt test. These abnormalities occurred in the absence of demonstrable changes in heart rate or blood pressure and were ameliorated through the use of compression stockings.

### Is POTS a cause of OI?

Although some forms of dysautonomia causes orthostatic instability accompanied by abnormal changes in heart rate and blood pressure, whether disorders of the autonomic nervous system are responsible for OI, and whether OI occurs in ME/CFS patients, remains controversial (46, 47, 53, 58, 59). Jones et al. (63) reported that orthostatic instability was similar in persons with CFS and control subjects recruited from the general Wichita population. Interestingly, persons with higher serum osmolarity had significantly higher rates of abnormal tilt responses than those with lower serum osmolarity, suggesting that delayed responses to head-up tilt tests may reflect hydration status (63). A reappraisal of primary dysautonomia as a factor in the pathogenesis of OI, with or without ME/CFS, may be warranted. Low-dose oral propranolol (20 mg) significantly attenuated tachycardia and improved symptoms in POTS (64). In the meantime, Ewan et al. (65) reported that the use of the sinus node blocker ivabradine led to dramatic improvements in both subjective and objective symptomatology, accompanied by a reduction in heart rate on standing, in a 15-year-old female patient with POTS. The use of this medication appears to improve not only tachycardia but also symptomatology, including fatigue, suggesting that tachycardia may not only be unnecessary for maintaining cerebral perfusion during standing as a compensatory mechanism but also triggers many symptoms, possibly through disturbances in the autonomic nervous system.

I have reported interesting findings of “chronotropic intolerance” in some patients with ME during standing tests, suggesting impaired sympathetic activation (66–68). In some ME subjects in those reports, failure to complete 10 min of standing without POTS was observed in some of the standing test(s), whereas success was associated with POTS on other occasions. The presence of POTS, with sufficient sympathetic activation, appears to be essential for maintaining orthostasis in these patients. In 2019, Garner and Baraniuk graded symptom intensity or severity of OI using a 0–20 anchored, ordinal scale in CFS patients (69). Although they had hypothesized that CFS subjects with POTS would have higher standing scores than those without POTS (non-POTS), they found that orthostatic tachycardia did not account for OI symptoms in CFS. Indeed, more CFS subjects had OI symptoms without postural tachycardia than had POTS, and the magnitudes of OI symptoms did not differ between POTS and non-POTS subjects in either the CFS or control groups. Therefore, POTS did not explain the OI symptoms in the CFS cohort. Further examinations will clarify the exact role of POTS in the pathophysiology of POTS.

### Therapeutic implications for OI in patients with ME

Elucidation of the pathophysiology of ME and OI may lead to better therapeutic strategies. Xenon-computed tomography blood flow studies demonstrated that CFS patients exhibit global cerebral hypoperfusion, with reduced absolute cortical blood flow across broad areas, particularly in the bilateral middle cerebral artery territories, compared with healthy controls (70). Impaired cerebral oxygenation due to reduced cerebral hemodynamics in young CFS with OI was suggested by findings from continuous measurements of cerebral oxygenated hemoglobin using near-infrared spectroscopy (71) during an active standing test. In addition, both systolic and diastolic blood pressures were lower in OI patients with and without CFS compared with control subjects. Newton et al. (72) have consolidated the evidence using 24-h ambulatory blood pressure monitoring that lower blood pressure occurs in CFS patients, and lower nighttime blood pressure appears to be a significant problem that may lead to enhanced diurnal variation. The putative mechanism by which OI and ME/CFS are triggered or caused in patients with a small heart and/or ME is shown in Figure 1.

Reasonable potentiation of cerebrovascular flow, without exaggerated activation or perturbation of the autonomic nervous system, may be necessary for effective treatment. However, administration of non-selective vasoconstrictive agents may cause a simple reduction in cerebral blood flow through increased cerebrovascular resistance, although intravenous infusion of phenylephrine, a sympathetic nerve α_1_ stimulator, has been reported to improve OI by producing significant peripheral vasoconstriction and venoconstriction effects in some OI patients (73).

A small heart with reduced cardiac performance due to decreased preload may be an important target for the treatment of CFS. Volume repletion through increased sodium intake or by treatment with fludrocortisones may theoretically improve OI and also symptoms of CFS by replenishing intravascular volume (37, 43, 46, 50, 56). Military anti-shock trousers, as well as elastic stockings that compress the lower extremities, may also be effective by potentiating venous return, resulting in increased cardiac output (59). Although ME/CFS patients are limited by the discomfort of an increased perception of exertion and excessive exertion may result in a “crash,” characterized by acute exacerbation of symptoms requiring prolonged bed rest, some evidence support the notion that an appropriately designed exercise program is beneficial (53, 74–76). Various triggers, including loss of appetite, diarrhea, and sweating, can cause dehydration accompanied by preload reduction, leading to further decreases in stroke volume and cardiac output, thereby impairing both systemic and cerebral circulation and exacerbating symptoms; therefore, they should be avoided or treated appropriately.

## Orthostatic intolerance in ME—update and paradigm shift

### Down-regulation of circulatory blood volume in patients with ME

A majority of the patients with ME/CFS have orthostatic intolerance (OI), which primarily restricts daily functional capacity and, in turn, quality of life (25, 37, 45, 50, 77, 78). A small heart or reduced left ventricular volume with reduced cardiac output has been reported to be common in patients with ME. The marked postural orthostatic tachycardia accompanied by OI, observed in many patients with ME, appears to be primarily a physiologic compensatory response to a smaller stroke volume on standing (45, 50, 77). In these patients, further preload reduction during standing, which causes impaired cerebral oxygenation because of reduced cerebral hemodynamics with or without dysfunctional circulatory autoregulation (71), is the condition that triggers the activation of the main regulatory systems for the circulatory blood volume, including the renin–angiotensin–aldosterone (R-A) and antidiuretic hormone (ADH) systems (Figure 1). The main regulators of circulatory blood volume regulators may be downregulated in ME patients.

Plasma levels of neurohumoral factors regulating circulatory blood volume were measured in patients with ME and compared with those in controls (79, 80). The echocardiographic examination revealed that the mean values for the left ventricular end-diastolic diameters, stroke volume index, and cardiac index, as well as the mean blood pressure, were significantly lower in the ME group than in the controls. The mean plasma renin activity was considerably lower in the ME group than in the control group. Both mean plasma aldosterone and ADH concentrations were significantly lower in the ME group than in the control group. Desmopressin (120 μg), a synthetic analog of arginine vasopressin, was orally administered for five consecutive days to 10 patients with ME and urinary osmotic pressure <500 Osm/kg H_2_O. In five patients (50%), symptoms of orthostatic intolerance during a 10-min active standing test were ameliorated, accompanied by a significant increase in urinary osmotic pressure and a decrease in heart rate. Furthermore, in five patients (50%), performance status scores for activities of daily living improved. Thus, both the R-A and ADH systems were downregulated despite reductions in cardiac preload and output in patients with ME. Desmopressin improved symptoms in half of the patients. These patients with low urinary osmotic pressure and favorable therapeutic effects with desmopressin appear to be associated with a mild or partial type of diabetes insipidus.

Despite the reduction in circulatory volume, impaired activation of the renin–aldosterone system, known as the renin–aldosterone paradox, has been reported in patients with OI and POTS, as well as in patients with ME (80, 81). The reason why the renin–aldosterone system fails to be activated remains to be elucidated. The hypothalamus–pituitary–adrenal axis is another system involved in aldosterone production. From the findings of low levels of cortisol in plasma and urine with insufficient response in corticotropin-releasing hormone and adrenocorticotropic hormone challenge test in patients with CFS, impaired activation of both the axis and sympathetic nervous system in response to excitatory stimuli has also been reported in the patients (82–84), suggesting structural or functional brain abnormalities. In addition, reduced activation of the ADH system in the hypothalamus–pituitary axis was observed in patients with ME. Both the R-A and ADH systems, the main neurohumoral regulatory systems for circulatory blood volume, appear to be impaired or downregulated probably due to central nervous system dysfunction or hypothalamus–pituitary–adrenal system. ADH is produced in the hypothalamus, carried into the pituitary gland through the axon, and released into blood circulation from the posterior lobe of the pituitary gland. Both non-osmotic and osmotic stimulation are important factors for ADH release in physiologic conditions (85, 86). Because the blood osmotic pressure was comparable between the ME group and the control group, apparently osmotic stimulation was not enhanced in ME. As for the non-osmotic control, the decrease in cardiac output due to reduced effective circulatory volume is usually believed to inactivate the tonic inhibition of baroreceptors on ADH release (87). Both high-pressure baroreceptors at the carotid sinus and aortic arch and low-pressure baroreceptors at the pulmonary veins and left atrium are involved in the inhibition of neurohormone release of ADH (81). This control mechanism process for the inactivation of the tonic inhibition of ADH release may be impaired in the central nervous system. No indication of relevant organic pathology in either the hypothalamus or hypophysis was found, suggesting a functional or metabolic dysfunction possibly based on neuroinflammation (88). Low plasma ADH concentrations have also been reported among adolescent CFS patients (89). Desmopressin, which acts on the vasopressin V_2_ receptors to promote antidiuresis, was administered orally to patients with ME. In half of the patients, with urinary osmotic pressures below 500 Osm/kg H_2_O, the OI symptoms were attenuated or improved in association with a significant increase in urinary osmotic pressure and a decrease in heart rate. Similar results in patients with POT syndrome were reported by Coffin et al. (90). Desmopressin therapy can be a possible effective treatment for patients with ME with low urinary osmotic pressure. Adequate water intake appears to be essential for the possible beneficial effects of desmopressin. In patients with favorable effects of desmopressin, the stroke volume index increased, whereas the cardiac index did not increase due to a considerable decrease in heart rate, suggesting sympathetic unloading. In several previous reports, a clear correlation was found between fatigue sensation and muscle sympathetic nerve activity during static contraction (83, 91). Furthermore, exhaustive incremental exercise provokes sustained increases in plasma noradrenaline levels that outlast the termination of exercise by several hours (92). Inappropriate sympathetic overactivity at rest, which represents a neural functional correlate of fatigue (83, 91), may have been ameliorated by desmopressin treatment in these patients. Obviously, further investigation in a larger number of patients, including estimates of water and salt intake and hormone levels, will be required to clarify the mechanism underlying the apparent impairment of activation of the R-A and ADH systems and the beneficial therapeutic effects of desmopressin in some patients.

Use of desmopressin (1-deamino-8-D-arginine vasopressin), a synthetic vasopressin (ADH) receptor agonist, has expanded beyond the treatment of diabetes insipidus in recent years. A low dose of desmopressin may exert favorable effects in patients with ME/CFS and a small heart by increasing circulatory blood volume. Potential side effects of desmopressin include hyponatremia, edema, and headache, as it increases free-water retention (90). Hyponatremia, in particular, which may lead to disturbances of consciousness, is a side effect that would be of significant concern with daily dosing. Given these safety concerns, a daily of 120 μg desmopressin would not be recommended for patients. Instead, a daily dose of 60 μg desmopressin would be recommended for safe use. This dose is substantially lower than that used for the treatment of patients with diabetes insipidus and may not require frequent blood monitoring of sodium level, although serum sodium concentration should be within the normal range prior to initiating or resuming treatment. Therapeutic use of this drug is contraindicated in patients with hyponatremia or a history of hyponatremia. Prior to considering desmopressin therapy in patients with both ME and small heart, secondary arginine vasopressin (ADH) resistance or renal diabetes insipidus due to specific causes such as lithium ingestion, hypercalcemia, and hypokalemia should be excluded. Anecdotal observations also suggest that 120 μg desmopressin can be used successfully as an occasional “as needed” medication to achieve acute improvement in symptoms. The majority of healthy subjects have plasma ADH level of ≥1.6 pg./mL (80, 88). Predictive biomarkers of urinary osmotic pressure of <300 mOsm/kg H_2_O and plasma ADH level of <1.6 pg./mL could identify responders before starting treatment.

Intravenous fluid repletion or fludrocortisone may exert an acute, favorable therapeutic effect through transient volume expansion or increased preload. However, termination of this procedure over several successive days would result in volume depletion or reduced preload, with a lack of or worsening of favorable effects due to an additional decrease in aldosterone (aldosterone escape) (93).

In conclusion, in patients with ME, both the R-A and ADH systems, the main blood volume regulators, are downregulated despite reduced cardiac preload and output. Furthermore, oral administration of desmopressin improved symptoms in half of the patients. Patients with urinary osmotic pressure below 300 mOsm/kg H_2_O, who are constantly observed at the clinic visits, may have a kind of or a mild type of diabetes insipidus and are worth trying oral administration of desmopressin.

### Paradigm shift to disequilibrium in the genesis of OI

Postural stability is necessary to allow the static balance critical for performing many daily activities. Recently, we have reported that postural instability or disequilibrium should be considered to be involved in the pathogenesis of OI in patients with ME/CFS (94–96) (Table 1). It appears to be of central vestibular origin, consistent with previously reported results of vestibular function tests in patients with chronic fatigue syndrome (97, 98). Disequilibrium is classified as static and kinetic. Our recent reports have suggested that static disequilibrium causes postural instability resulting in OI (94–96, 99), while kinetic disequilibrium often results in gait disturbance (100–102). Kinetic disequilibrium observed during the tandem gait test in patients with ME has three distinct manifestations: direction predominance, opposite-direction step-out between forward and backward steps, and disequilibrium worsened after turning around, suggesting a vestibular nerve system deficit. Usually, proprioceptive sensation is preserved in ME patients, suggesting no disequilibrium due to spinal sensation damage. In addition, these patients have no limb ataxia, suggesting no disequilibrium due to cerebellar damage.

**Table 1:** Relationship between orthostatic intolerance and static or kinetic disequilibrium in 160 ME patients (53 male and 107 female patients with a mean age of 37 ± 12 years).

Disequilibrium	Number of patients (%)	Failure of 10-min standing
Static disequilibrium	40 (25%)	22 (55%)
Kinetic disequilibrium	71 (44%)	27 (38%)
Any	72 (45%)	27 (38%)
None	88 (55%)	0 (0%)

Static disequilibrium: Unstable standing with their feet together and eyes open and/or closed. Kinetic disequilibrium: Unstable tandem gait with turn and return.

Static balance and postural stability are regulated by a postural adjustment system (101, 102). Multiple types of sensory information, such as somatosensory, visual, and vestibular sensations, are processed by various brain areas within the multisensory cortex and then in the corticocortical vestibular network to achieve adaptive postural control. The automatic process of postural control is termed postural reflex and includes head-eye coordination accompanied by appropriate body segment alignment (101, 102). This control is mediated by descending pathways from the brainstem, with cooperation between the vestibulospinal, reticulospinal, and tectospinal tracts contributing to the process. The visual sensory-mediated system can compensate for central vestibular function to maintain postural control when the abovementioned system is impaired in the brain cortex. Visual information from the eyes is projected directly to the vestibular and oculomotor nuclei in the midbrain, stimulating the head and external oculomotor muscles and helping maintain postural stability, thereby supporting vestibular function in cooperation with the cerebellum. Visual compensation is therefore likely to be essential for maintaining postural stability in the presence of vestibular deficits (101, 102), explaining why many patients with ME lost balance during the Romberg test when their eyes were closed. Regulating kinetic stability is usually more difficult under locomotive conditions than stability under static conditions, particularly during tandem gait and 180° turns, even with eyes open. Indeed, my recent report indicated that most patients with a positive Romberg test or static disequilibrium have a positive tandem gait test or kinetic disequilibrium (99).

**Figure 2:**
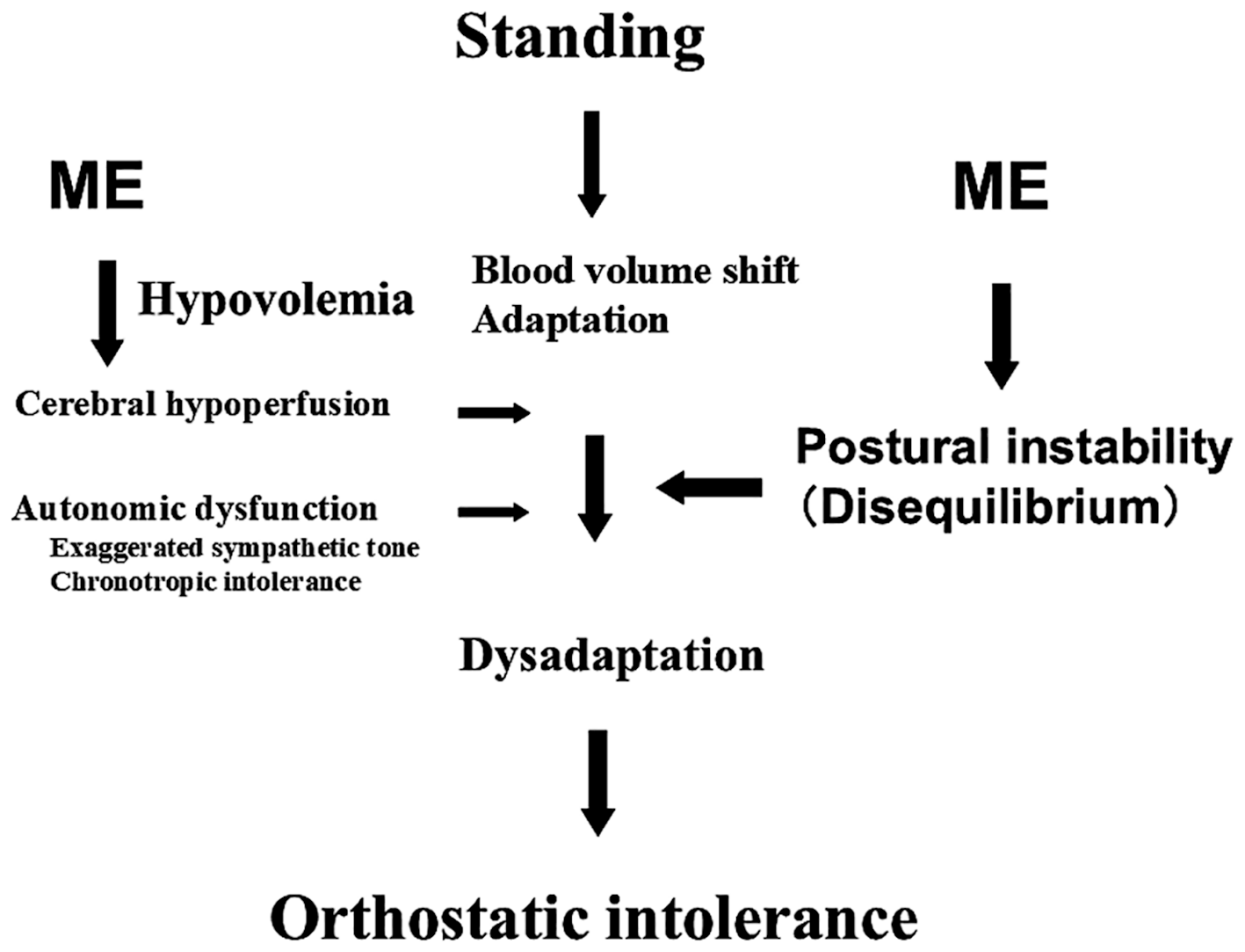
Paradigm shift of the mechanism for OI. Disequilibrium is included. ME: myalgic encephalomyelitis.

The conventional active 10-min standing test and neurologic examination revealed that the majority of patients with ME who could not stand for 10 min demonstrated disequilibrium, as evidenced by their difficulty with the Romberg test (standing with feet together and eyes closed) or static disequilibrium (Table 1) (94–96, 99). In addition, the majority of patients with ME and accompanying disequilibrium failed to complete the 10-min standing test, whereas failure to complete the test was extremely rare in ME patients who did not exhibit disequilibrium, suggesting the etiologic role of disequilibrium or truncal ataxia in OI (Table 1). Step-out, misstep, and/or body sway with the whole head displaced from the vertical line of gravity at the midpoint between both feet were deemed positive for kinetic disequilibrium and static disequilibrium (99). Similar body sway was frequently observed during the active standing tests in patients with disequilibrium (99). Instability associated with neurally mediated or reflex syncope during neurologic examinations can be easily differentiated from disequilibrium, as these patients are usually unable to maintain a standing position and may crouch down with bradycardia, hypotension, pallor, nausea, and even disturbance of consciousness during the attack. Moreover, the review of the records of the patient subgroup at their second visit revealed that some patients with disequilibrium who could not complete the test had been able to complete it and exhibited no disequilibrium at their first visit, suggesting that OI and disequilibrium developed concurrently during the interim period. In the meantime, OI improved in association with the amelioration of disequilibrium following favorable therapy with oral minocycline (103, 104) or repetitive transcranial magnetic stimulation (rTMS) (99, 105) in the patients. Disequilibrium in association with postural reflex dysfunction appears to be strongly associated with the expression of OI. Our results indicate that disequilibrium or postural reflex dysfunction is, in fact, causal for OI and is more related to OI than POTS in patients with ME/CFS (Figure 2). In addition, objective quantitative data concerning disequilibrium have been reported in the patients. Serrador et al. (106) examined balance using posturography in people with CFS during static balance tasks and have reported that they have poor postural control associated with poor physical health. Hayes et al. (107) examined postural sway using isoinertial measurement units during a 30-s static stand test. They reported that both people with long COVID-19 and people with ME/CFS have similarly impaired balance and physical capacity.

rTMS is a newly developed neuromodulatory procedure and was reported to be studied as a treatment for a wide variety of neurological as well as psychiatric disorders, such as stroke and depression (108, 109). A recent pilot study by Kakuda et al. (110) revealed that general fatigue symptoms were subjectively alleviated in patients with CFS after rTMS was applied over the dorsolateral prefrontal cortex (DLPFC). However, only a few patients were included. According to recent reports, rTMS of the primary motor cortex (M1) can have pain-relieving effects in patients with neuropathic pain or fibromyalgia (111–113), which is often comorbid with ME/CFS (2–4). In addition, a growing amount of evidence indicates that the left prefrontal cortex is involved in pain modulation (114, 115).

Treatment with rTMS to both the left DLPFC and M1 was effective in alleviating various symptoms, particularly OI and disequilibrium, and improving activities of daily living in patients with ME (105). The most favorable outcome of treatment was the alleviation of disequilibrium associated with the resolution of OI. Disequilibrium or postural reflex dysfunction is an important cause of OI. Treatment with rTMS can be considered as a therapeutic option. The corticovestibular network comprising the vestibular nucleus in the brainstem, several cortical vestibular nuclei, several midbrain regions, and the cerebellum is distributed throughout the brain and exhibits a high degree of functional connectivity (102, 116, 117). None of the major brain regions of the vestibular system were included in the targeted sites for rTMS in the studies. rTMS probably recovered or re-strengthened network function connecting through the brain stem, the brain cortex, and the cerebellum, as well as the midbrain, recovering total central vestibular function.

A typical case is shown in Supplementary Videos S1–S3.

In those studies (99, 105), rTMS treatment was applied to both the DLPFC and M1 in all participants. Consequently, the effect of this treatment could not be evaluated individually for the DLPFC and M1. In addition, the long-term effects of rTMS treatment remain to be determined. In this study, rTMS was applied to fixed sites in the left DLPFC and the left M1, irrespective of right- or left-handedness, and it worked. Although which is the better targeted site for rTMS, right or left DLPFC, remains to be determined, the procedure can work irrespective of right- or left-handed.

Patients whose disequilibrium was alleviated by rTMS could complete the 10-min standing test, demonstrating improved OI and suggesting that postural stability is essential for maintaining an erect position. Dysfunction of postural reflex, or disequilibrium, appears to play an important role in the etiology of OI. OI appeared to be caused primarily by neurologic abnormalities in the central nervous system in majority of the patients with ME rather than being of cardiovascular origin. OI is a primary factor in restricting daily functional capacity (77). It has been reported that patients with disequilibrium had higher performance status scores, indicating the degree of restriction of activities of daily living, than those without it, suggesting more severely restricted activities of daily living (95, 96, 99).

### Active standing test or head-up tilt test

The head-up tilt test is often used to diagnose OI. Compared with head-up tilt tests, active standing tests are more conventional and better preserve leg muscle pump function, although head-up tilt tests can more readily induce reflex mediated or neurally mediated syncope. However, head-up tilt testing can mask the handicap with disequilibrium. Unlike the active standing test, the tilt test, in which the subjects’ bodies are fully fixed on the tilting board, can detect OI due to only circulatory problems, with or without autonomic dysregulation, but not OI due to disequilibrium. Active standing tests are apparently more suitable than head-up tilt tests for detecting OI in patients with ME or possibly long COVID-19. Recently, a study using neurologic evaluations and active standing tests has reported that disequilibrium, rather than POTS, is the primary determinant of OI in patients with long COVID-19 (118). Patients with disequilibrium may require increased effort to maintain an upright posture, resulting in exaggerated sympathetic activation and leading to severe fatigue or exhaustion.

## Conclusion

It is necessary to consider the involvement of postural instability or disequilibrium, which may be possibly related to central vestibular dysfunction in the pathogenesis of OI in patients.

## Supplementary material

SUPPLEMENTARY VIDEOS S1–S3 Effects of rTMS on disequilibrium. A 30-year-old male patient with long COVID-19 and myalgic encephalomyelitis, and a disease duration of 7 months, who was unable to continue standing after 7 min during the standing test (OI). He received oral minocycline treatment, but it did not work. The left movie shows the patient before rTMS treatment. He was unable to stand stably with both feet together and eyes open, exhibited considerable body sway with his eyes closed, had unstable single-leg stance, and misstepped in tandem gait, demonstrating disequilibrium. In contrast, the right movie shows the patient after rTMS to the left dorsolateral prefrontal cortex and the primary motor cortex. He recovered from OI and could stand stably with both feet together and eyes open and closed. He also exhibited stable single-leg standing and a stable tandem gait.
